# Characterization of Early Cortical Neural Network Development in Multiwell Microelectrode Array Plates

**DOI:** 10.1177/1087057116640520

**Published:** 2016-03-29

**Authors:** Ellese Cotterill, Diana Hall, Kathleen Wallace, William R. Mundy, Stephen J. Eglen, Timothy J. Shafer

**Affiliations:** 1 Department of Applied Mathematics and Theoretical Physics, University of Cambridge, Cambridge, UK; 2 Office of Research and Development, National Health and Environmental Effects Research Laboratory, U.S. Environmental Protection Agency, Research Triangle Park, NC, USA

**Keywords:** neurological diseases, cell-based assays, toxicology, membrane potential

## Abstract

We examined neural network ontogeny using microelectrode array (MEA) recordings made in multiwell MEA (mwMEA) plates over the first 12 days in vitro (DIV). In primary cortical cultures, action potential spiking activity developed rapidly between DIV 5 and 12. Spiking was sporadic and unorganized at early DIV, and became progressively more organized with time, with bursting parameters, synchrony, and network bursting increasing between DIV 5 and 12. We selected 12 features to describe network activity; principal components analysis using these features demonstrated segregation of data by age at both the well and plate levels. Using random forest classifiers and support vector machines, we demonstrated that four features (coefficient of variation [CV] of within-burst interspike interval, CV of interburst interval, network spike rate, and burst rate) could predict the age of each well recording with >65% accuracy. When restricting the classification to a binary decision, accuracy improved to as high as 95%. Further, we present a novel resampling approach to determine the number of wells needed for comparing different treatments. Overall, these results demonstrate that network development on mwMEA plates is similar to development in single-well MEAs. The increased throughput of mwMEAs will facilitate screening drugs, chemicals, or disease states for effects on neurodevelopment.

## Introduction

Microelectrode array (MEA) recordings are a useful tool to study the activity of networks of interconnected neurons, both in vitro and in vivo. In vitro, neural networks on MEAs demonstrate many characteristics of intact neural networks; this includes extracellular recordings of action potentials (“spikes”) and groups of action potentials (“bursts”) simultaneously from multiple points in the network.^1^ The spontaneous activity in these networks exhibits pharmacological responsiveness and plasticity.^2–5^ Thus, primary cultures of neural networks on MEAs have been widely utilized to study neurophysiology, neuropharmacology, and neurotoxicology (for review, see Johnstone et al.^6^). In addition, the ontogeny of network activity on MEAs has been described by numerous different laboratories.^4,7–10^ Until recently, however, the throughput of MEA devices has been limited, such that it was not possible to study more than a small handful (e.g., four to six) of networks at a time.

Recently, two manufacturers of MEA devices have introduced multiwell MEA (mwMEA) devices, which allow for recordings to be made from 12–96 wells simultaneously, with 8–64 electrodes/well. The increase in throughput offered by mwMEA devices expands the capabilities of MEA systems, allowing for drug and toxicant screening.^11,12^ Further, when combined with cultures that have undergone genomic manipulation^13,14^ or with patient-derived inducible pluripotent stem cells,^15,16^ mwMEA recordings have been used to describe how network function is affected by disease states. Finally, mwMEAs offer the ability to screen large numbers of chemicals for potential effects on developing networks.^17,18^ Given the significant public concern over the potential role of chemicals in neurodevelopmental diseases,^19^ the study of chemical effects on the early stages of neural network ontogeny using mwMEAs offers a functional measure for developmental neurotoxicity hazard characterization.

However, in order for such studies to take place, the basic development of activity in networks on mwMEAs needs to be described in detail. In lower-throughput MEA systems, neural network development has been demonstrated to transition from low activity at early developmental ages (e.g., the first week in vitro) to one of coordinated bursting, network spikes, and synchrony at later time points (e.g., the second week in vitro and beyond). While it is expected that such properties will be retained in multiwell systems, it remains to be demonstrated, and the time-course, variability, and other characteristics defined. Further, while single-well MEAs contain ~60 microelectrodes, only 12-well mwMEA plates have an equivalent number (64/well), and the extent to which network properties can be defined with fewer electrodes has not been determined.

The present studies describe the development of networks of mixed primary cortical cultures in 48-well mwMEA plates containing 16 microelectrodes/well. These cultures were prepared from newborn rat cortex and contain excitatory and inhibitory neurons as well as glia.^20,21^ Over the first 2 weeks in vitro, the neurons extend axons and dendrites,^22^ form synapses,^23^ and, in single-well MEA systems, develop spontaneous network activity.^17^ The present studies characterized the early ontogeny of activity of these cultures in mwMEAs by describing the firing, bursting, synchrony, and network spike properties over the first 12 days in vitro (DIV), as previous studies with this culture model have shown that a significant change in the rates and patterns of activity occurs over this time frame. Further, we sought to evaluate the utility of analysis of multiple features of network activity as a method to determine the ability of classification approaches to distinguish between cultures under different conditions (e.g., control vs drug/toxicant treatment, different ages, or genetically modified vs wild-type).

## Methods

### Experimental Protocol

#### Cell culture

All procedures using animals were approved by the National Health and Environmental Effects Laboratory Institutional Animal Use and Care Committee. Primary cultures were prepared from the cortex of 0–24 h old rat pups as described previously.^11,20,21^ Cells were plated (1.5 × 10^5^ cells in a 25 µL drop of media) onto the surface of 48-well MEA plates (16 electrodes/well) that had been precoated with polyethylenimine (PEI) and laminin as previously described.^11^ The resultant cultures contain excitatory and inhibitory neurons and glia (**Suppl. Fig. 1**).

#### MEA recordings

Spontaneous network activity was recorded using Axion Biosystems Maestro 768 channel amplifier and Axion Integrated Studios (AxIS) v1.9 (or later) software. The amplifier recorded from all channels simultaneously using a gain of 1200× and a sampling rate of 12.5 kHz/channel. After passing the signal through a Butterworth band-pass filter (300–5000 Hz), online spike detection (threshold = 8× rms noise on each channel) was done with the AxIS adaptive spike detector. On DIV 5, 7, 9, and 12, plates were placed into the Maestro amplifier and allowed at least 5 min to equilibrate, after which at least 15 min of activity was recorded. As the majority of these plates were used on or around DIV 14 for other experiments, short equilibration and recording times (~30 min total) were selected to minimize the potential impact of repeated removal of cells from the incubator over the time period during which activity was developing. All recordings were conducted at 37 °C, and since development of activity was being studied, there were no a priori thresholds for minimum numbers of active electrodes for inclusion of a well in the data set.

### Data

Recordings were made from 656 wells across 16 MEA plates from 15 primary cortical cultures at DIV 5, 7, 9, and 12 for a total of 64 “plate recordings.” A recording of one plate at DIV 12 was missing from our data set, and one recording at DIV 7 was also excluded from the analysis, as its mean firing rate (MFR; 1.2 Hz) was greater than 2 SD above that of the other DIV 7 plates (mean = 0.6 Hz, SD = 0.3). This resulted in a total of 62 plate recordings, with a total of 2976 well recordings, used in our analysis. Activity was usually recorded for 15–30 min; only the last 15 min of each recording was analyzed. Features related to spikes, bursts, network spikes,^24^ and correlations^25^ were extracted in the R programming environment v3.0 using two open-source R packages, SJEMEA and MEADQ, and compiled into a well-level data set. Bursts were detected using an implementation of the MaxInterval method by Neuroexplorer, with the following threshold parameters: maximum interspike interval (ISI), 0.25 s; maximum beginning ISI, 0.1 s; minimum interburst interval (IBI), 0.8 s; minimum burst duration, 0.05 s; and minimum number of spikes in a burst, 6. Network spikes were identified by dividing the recording period into 3 ms bins and determining the number of electrodes in the well that fired at least one spike during each bin; the minimum threshold for a network spike was for spike activity to be present on at least five electrodes in a given time bin. Correlation was measured using the spike time tiling coefficient, which was defined as

$$STTC=\frac{1}{2}\left(\frac{P_{1}-T_{2}}{1-P_{1}T_{2}}+\frac{P_{2}-T_{1}}{1-P_{2}T_{1}}\right)$$

where P1 is the proportion of spikes on electrode 1 that occur within ±Δt of a spike on electrode 2, and T1 is the fraction of the total recording time that lies within ±Δt of a spike on electrode 1. P2 and T2 are the equivalent values on electrode 2. Data files generated by AxIS were converted into HDF5 file format;^26^ HDF5 files, scripts to generate the features, and related R objects are stored in a public repository (http://github.com/sje30/EPAmeadev). The goal of establishing a public data set is to allow full reproducibility of our analysis and/or to allow novel analyses to be conducted.

### Developmental Analysis

Twelve features were chosen to describe the culture activity, which are summarized in **Table 1**. For all features, the plate value was taken as the median of all nonzero well values on the plate (zero values were ignored).

**Table 1.** Features Used in Our Analysis and a Brief Description of How They Were Calculated.

Feature	Description
MFR	The MFR on each electrode was calculated. The well value was the median value of all active electrodes.
Burst rate	The number of bursts per minute on an electrode was calculated. The well value was the median value from all electrodes that exhibited bursting behavior.
Burst duration	The mean duration of all bursts on an electrode over the recording period was calculated. The well value was the median value from all electrodes that exhibited bursting behavior.
Fraction of bursting electrodes	An electrode was classified as bursting if the burst rate on the electrode was at least one per minute. The well value was the number of electrodes classified as bursting as a fraction of the total number of active electrodes on the well.
Within-burst firing rate	The mean firing rate within all bursts on an electrode was calculated. The well value was the median value from all electrodes that exhibited bursting behavior.
Percentage of spikes in bursts	The number of spikes on an electrode classified as being within bursts divided by the total number of spikes on the electrode. The well value was the median value from all electrodes that exhibited bursting behavior.
Coefficient of variation (CV) of IBI	The ratio of the standard deviation to the mean of the length of all IBIs on an electrode. The well value was the median value from all electrodes that exhibited bursting behavior.
CV of within-burst ISIs	The ratio of the standard deviation to the mean of the length of all ISIs within bursts on an electrode. The well value was the median value from all electrodes that exhibited bursting behavior.
Network spike rate	The well value was the number of network spikes on the well per minute of the recording period (see Methods section for definition of a network spike).
Network spike duration	The duration of a network spike was defined as the length of time during which the number of active electrodes on the well exceeded the threshold value (5). The well value was taken as the median duration of all network spikes on the well during the recording period.
Network spike peak	The maximum number of active electrodes during each network spike. The well value was taken as the median peak value of all network spikes on the well during the recording period.
Mean correlation	The correlation between every pairwise combination of electrodes on a well was calculated using the spike time tiling coefficient^25^ with ∆*t* = 50 ms (see Methods section for definition). The well value was the mean of the pairwise correlations between all distinct electrodes on the well.

### PCA

We performed principal components analysis (PCA) using the R package FactoMineR^27^ using all wells and all 12 features. Two PCAs were performed. The first PCA was conducted using data in which a well constituted one observation, while the second PCA was conducted using data in which a plate median constituted an observation. For each PCA, the 12-dimensional feature vector was projected down onto the plane created by the first two principal components dimensions. The purpose of the projection was to visually assess the level of differentiation among the four ages. A scree plot was made to describe the cumulative percent of variation explained by the use of additional principal components to describe the data. The scree plots aid in quantifying the extent to which data may be well represented with fewer dimensions.

### Classification

Classification was performed to understand whether and to what extent the features chosen above could distinguish between networks with different characteristics (e.g., control vs compound treated). Since this data set did not contain networks treated with compounds, our classification examined the ability of the chosen features to discriminate between networks of different ages. Two classification techniques, random forests and support vector machines (SVMs), were used to predict the age of each well based on the 12 features used in our analysis. In some cases, due to the low number of electrodes on a well, lack of bursting, or lack of network spike activity, some feature values were missing; this was particularly evident at early DIV. For classification purposes, for those wells with no bursts, the within-burst firing rate and burst duration were set to zero. Similarly, the network spike peak and duration were set to zero for all wells that exhibited no network spikes over the recording period. Any wells that had null values for the remaining features, namely, correlation, CV of IBI, and CV of within-burst ISI, were excluded from the classification. This resulted in 370/2976 well recordings, or approximately 12.4% of the total wells, being excluded from the classification.

Initially, classification was performed on the remaining data using a random forest model and all 12 features. The relative importance of each of the features was determined based on the amount they reduced the Gini index. Next, SVMs were used to examine the classification accuracy obtained by using various subsets of the total 12 features. This four-class classification problem was addressed using the “one-against-one” approach,^28^ which involved building six binary SVMs, one for each pairwise combination of ages. Each of these SVMs were then used to binarily classify every data point, and the class to which each point was most frequently assigned across the six SVMs was taken as its correct class. A radial kernel was chosen over linear and polynomial kernels for the SVMs because of its superior classification accuracy on our data. The optimal regularization parameter values for the radial kernel were found by conducting a grid search across a range of values using 10-fold cross-validation on the entire data set. The parameters were chosen as those that maximized the cross-validation accuracy, which were $\gamma =\frac{1}{10}$ and C = 10.

In both types of classification, two-thirds of the data were used as a training set and the remaining third used to test the classification accuracy of the model. The classification was repeated 100 times using random choices of the training and test sets in each iteration, and the classification accuracy averaged over the 100 repetitions.

## Results

### Developmental Profile

**Figure 1.**
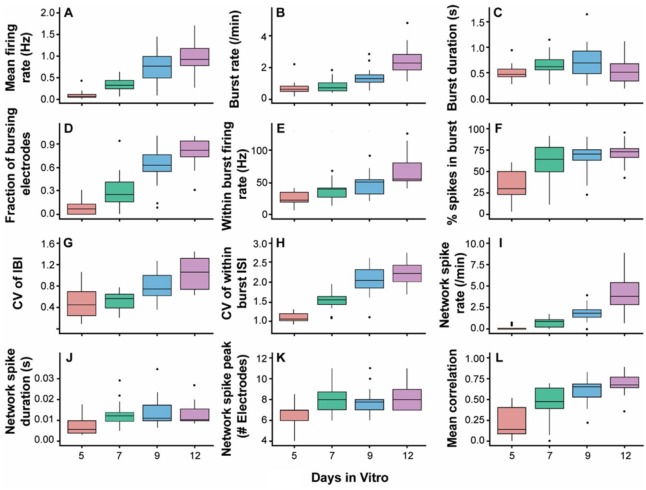
Mean firing (**A**) and burst rates (**B**) increase with development. Box plots showing median and interquartile range are shown for *n* = 16 plates. (**C**) Burst duration. (**D**) Fraction of bursting electrodes. (**E**) Within-burst firing rate. (**F**) Percentage of spikes in bursts. (**G**) CV of IBI. (**H**) CV of within-burst ISI. (**I**) Network spike rate. (**J**) Network spike duration. (**K**) Network spike peak. (**L**) Mean pairwise correlation.

On DIV 2, only rare, individual spikes were recorded (data not shown). Spontaneous activity in the neural networks arose and could be reliably recorded beginning on DIV 5 (**Suppl. Fig. 2**). Activity as assessed by most of the parameters used here increased with DIV. In particular, not only did spiking increase with time, but the organization of spiking into bursts and correlated activity across the network (**Suppl. Fig. 2**) also increased with DIV. Quantification of the changes in activity over development was achieved using a selection of 12 measures (**Fig. 1**), which were used to describe activity at the level of the entire well or individual electrodes, which were then aggregated into well-level values by taking the median.

#### Spontaneous firing rate

In general, activity increased over development, with the mean firing and burst rates both monotonically rising with increasing DIV (**Fig. 1A**,**B**).

#### Bursting activity

A clear increase in bursting activity with increasing culture age was also observed. Although burst duration did not show strong developmentally related changes (**Fig. 1C**), the fraction of bursting electrodes, within-burst firing rate, and percentage of spikes occurring within bursts all increased over development (**Fig. 1D–F**). The CV of IBIs and within-burst ISIs also increased with development, indicating a decrease in the regularity of these features (**Fig. 1G**,**H**).

#### Synchronous activity

The synchrony of activity within each individual well on a plate was examined using a feature called network spikes. Network spikes were defined as short time intervals in which the number of active electrodes on the well exceeded a threshold value, and their rate, duration, and peak number of active electrodes were quantified for each plate (**Fig. 1I–K**). The frequency of network spikes increased with increasing developmental age. To a lesser extent, an increase in the network spike peak (the maximum number of electrodes active during a network spike out of a possible 16) was also observed across development.

As another measure of network synchrony, we calculated the mean of all pairwise correlation coefficients for all electrodes in a well, using the spike time tiling coefficient.^25^ Correlations strengthened over development, particularly at early ages (**Fig. 1L**).

### PCA

**Figure 2.**
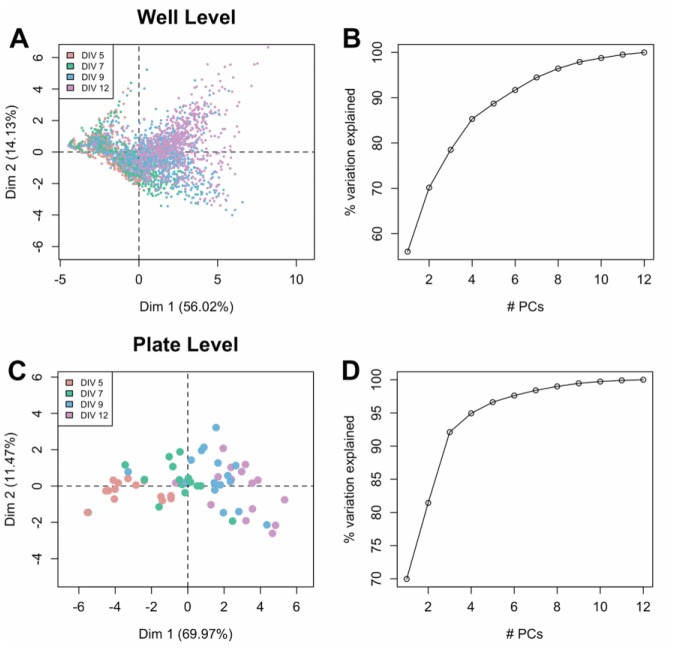
(**A**) Well-level PCA projection of 12-dimensional feature vectors onto PC dimensions 1 (*x* axis) and 2 (*y* axis). Each dot represents a well, colored by DIV of recording. Rough ordering from youngest (red, DIV 5) to oldest (purple, DIV 12) wells is apparent in change of colors along the positive direction. (**B**) Scree plot displays percent variance explained by the number of PC dimensions. (**C**) Plate-level PCA projection of plate medians onto PC dimensions 1 (*x* axis) and 2 (*y* axis). As in the top, rough ordering of observations by DIV is apparent in the red-to-purple transition along the *x* axis. (**D**) Scree plot of plate-level PCA. Compared to the well-level PCA scree plot, a larger amount of variation is captured in the first two PC dimensions, indicating that taking the plate median reduces variability.

A PCA was undertaken to visualize the level of differentiation among the four culture ages. The wells projected onto the first two PC dimensions (**Fig. 2A**) show a stochastic organization starting from the earliest age (red, DIV 5) progressing through to the oldest age (purple, DIV 12). The progression in age is roughly aligned with the first PC dimension, which accounts for more than 50% of the variation (**Fig. 2B**). To quantify the relationship between PC dimension 1 and culture age, PC1 was regressed against culture age. The linear model results showed that PC1 increases with increasing culture age (*p* value of slope < 0.001; **Suppl. Fig. 3**). This means that the principal mode of variation corresponds to the difference in ages of the cultures. Moreover, all factor loadings are positive on the first PC dimension, meaning that an increase in PC1 is associated with an increase in all 12 variables. Another salient aspect of the PC projection is that variation appears smaller at earlier ages. Similarly, the projection of the plate medians onto the first two PC dimensions yields a rough segregation by DIV. As in the well-level PCA, at the plate level, DIV are aligned with the first PC dimension (**Fig. 2C**), revealing a consistent age-related characteristic of the data. A greater percentage of variability is captured by the first PC dimension (67%; **Fig. 2D**) compared with the well-level PCA, related to the fact that taking the median reduces well-to-well variability. Both PCAs display sufficient visual differentiation between observations by DIV that a more thorough quantification of this separation is warranted through classification techniques.

### Classification

Classification techniques were used to determine the degree to which the recordings could be separated into their ages using the features specified above. First, a random forest model was built and used to predict the age of each well, using the 12 features from our analysis. The model was built using two-thirds of the data as a training set and its accuracy determined by using the remaining one-third of the data as a test set. When used to predict the age of each well from the four possible ages, the accuracy of the random forest model, averaged over 100 trials, was approximately 72% (compared to the 25% accuracy that could be expected by chance).

From these random forest models, we were also able to determine the relative importance of each of the features in driving the classification (**Table 2**, “Importance” column). The two most important features were those measuring coefficients of variation, namely, the CV of within-burst ISI and the CV of IBI. In our developmental analysis, these two features both exhibited a monotonically increasing trend with age.

**Table 2.** Classifier Performance at Predicting the Age of Arrays.

Feature	Importance	Accuracy %
CV of within-burst ISI	1.00	49.2
CV of IBI	0.70	58.3
Network spike rate	0.50	62.0
Burst rate	0.49	65.0
Burst duration	0.44	66.0
% spikes in bursts	0.39	68.3
Correlation	0.36	69.5
Firing rate	0.35	71.4
Within-burst firing rate	0.31	72.7
Bursting electrodes	0.22	73.0
Network spike duration	0.18	73.5
Network spike peak	0.09	73.4

Features are listed in decreasing order of importance, based on the importance score in column 2, derived from random forest classification and normalized to the top score. The value in each row *n* = 1, …, 12 of column 3 is the mean percentage of correct classifications using the top *n* features in the SVM model. For example, row 4 shows that the classifier was 65.0% accurate at predicting age using the top four features.

Next, we used SVMs to quantify the degree to which recordings could be classified correctly by age when only a subset of our features was used. The SVM classifier, built using the same proportion of training and test sets specified above, had a slightly higher level of accuracy, of approximately 73%, compared to the random forest model using all 12 features. Using the ordering of feature importance found above, we were then able to analyze how prediction accuracy varied as the number of features was reduced. **Table 2** shows the performance of the SVM as the number of features used in the classification was gradually reduced from 12, in the bottom row, to just 1, CV of within-burst ISI, in row 1. In general, we found that the classification accuracy remained high (~70% or higher) as the number of features was reduced. However, four features (burst rate, network spike rate, CV of IBI, and CV of within-burst ISI) were required to maintain a prediction accuracy of ≥65% (**Table 2**, “Accuracy %” column).

We used a similar method to examine the extent to which each pair of ages of arrays could be separated using classification techniques. In this case, rather than using all of the data in the classification, the SVM classifier was built separately on each pairwise combination of ages. The classifier was most accurate in distinguishing arrays with large differences in age, for example, DIV 5 and 12 arrays, for which only the top feature, CV of within-burst ISI, was required to achieve almost 92% prediction accuracy (**Table 3**). Classification performance was poorest for pairs of arrays in which the age difference was low. For example, the prediction accuracy for distinguishing DIV 9 from DIV 12 arrays was only just above chance when using one feature. Using all features improved the ability to distinguish between closely related ages to ~82%–83%, which is well above chance (**Table 3**).

**Table 3.** Classifier Performance at Predicting the Age of Arrays for Each Pairwise Combination of Ages (DIV).

	Accuracy %					
Feature	5 vs 7	5 vs 9	5 vs 12	7 vs 9	7 vs 12	9 vs 12
CV of within-burst ISI	75.0	87.5	91.8	69.9	78.1	57.4
CV of IBI	77.4	89.5	93.6	76.8	85.5	64.7
Network spike rate	79.3	90.3	95.5	79.6	88.0	68.7
Burst rate	79.3	90.3	95.3	81.0	88.4	72.8
Burst duration	79.7	90.8	95.3	81.0	88.6	74.0
% spikes in bursts	81.6	91.3	95.6	81.5	90.4	76.4
Correlation	82.2	92.1	95.6	82.3	90.9	77.1
Firing rate	82.3	91.7	95.7	82.3	90.9	80.2
Within-burst firing rate	84.2	92.7	96.2	82.4	91.3	81.2
Bursting electrodes	83.6	92.5	96.2	82.6	91.8	81.5
Network spike duration	83.7	92.9	96.8	82.9	92.8	82.0
Network spike peak	82.2	92.5	96.8	82.6	93.0	82.1

Features are listed in decreasing order of importance, and the value in each row *n* = 1, …, 12 is the mean percentage of correct classifications using the top *n* features, as described in **Table 2**.

### How Many Wells Are Needed?

In our experiments, we have used all 48 wells on a plate as replicates of the same experimental condition. This is a conservative way of using the multiwell array, and an alternative, higher-throughput approach might be to use different wells for different experimental conditions. However, there is inevitably a trade-off between the number of experimental conditions tested and the number of replicate recordings of conditions when assigning conditions to wells on a plate.

**Figure 3.**
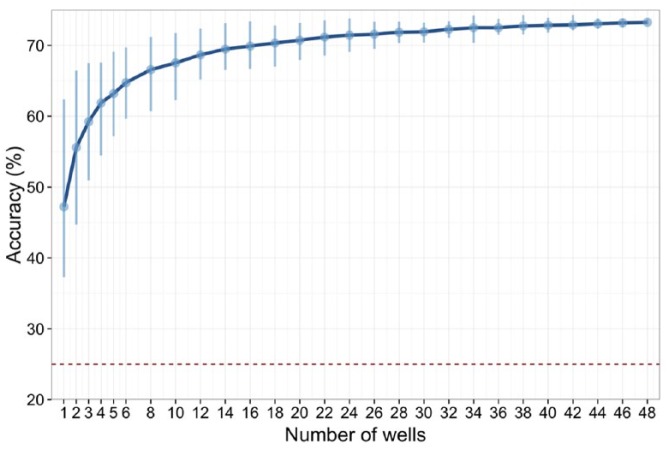
Accuracy of predicting the age of each well by sampling *n* ≤ 48 wells on each plate. Dark blue line shows the mean accuracy, while the vertical lines show the minimum and maximum accuracy over 100 trials with random choices of wells. For *n* = 48, the error bars indicate the small variability in classification due to the partitioning of data into training and test sets. The red dotted line indicates baseline level of performance (25%) for a classifier.

We therefore sought to investigate how robust our results were if fewer wells were used to form a signature of activity at a given age. Intuitively, we expected that with fewer wells, we would get less reliable signatures of activity, and hence poorer classification. Rather than run experiments where fewer wells were used, we simulated the experiments by randomly removing all data for a given number of wells on each of the 16 plates, and then repeated our classification tests to see how well each age could be discriminated. **Figure 3** shows that classification accuracy remained above 60% with as few as four wells per plate. With 16 wells (one-third of normal), the classifier accuracy is close to the stable value. For our particular question then of discriminating the four ages, we could get reliable results using one-half (24 wells) or one-third (16 wells) of the data that we generated for each plate.

## Discussion

The present results describe the ontogeny of network activity in 48-well MEA plates during the first 12 DIV. The results demonstrated a rapid ontogeny of spiking, bursting, synchrony, and network spiking activity over this period of time, which is similar to the ontogeny of activity in single-well MEAs. Furthermore, these results demonstrate that by considering multiple parameters of network firing, bursting, and synchrony properties, PCA and classification methods can be used as reliable predictors of network age at both the plate and well levels. These results demonstrate that the neural network ontogeny on mwMEAs offers, relative to single-well systems, a high-throughput approach to study network development and its perturbation by drugs, chemicals, and disease.

Previous studies of cortical and hippocampal network ontogeny have demonstrated that activity begins with random, single spiking on a single or few channels, and over a period of 2–3 weeks in vitro progresses to bursting activity that becomes more synchronous with time.^4,7–10,29^ This is accompanied over time by the emergence of network bursts. Similar to previous data from our laboratory using single-well MEA “chips” where cells were seeded at a high density,^17^ the ontogeny of spiking and bursting activity occurred rapidly within the first 2 weeks in vitro, specifically between DIV 5 and 12 in the present study. Our data are consistent with those of other laboratories that have reported initiation of spiking activity as early as 3–6 DIV.^7,30^ While our data describe the initial stages of the ontogeny of network activity, we did not examine ages beyond DIV 12 in the current study, and cannot rule out that some features examined may continue to “mature” with additional time in culture. Further, the ontogeny of network development in the culture used here does occur earlier than has been reported by other laboratories.^18,31–33^ Factors that may influence these differences include the age at which the cells were isolated (postnatal day 0 here vs embryonic day 18 in other studies), as well as the plating density (150,000 cells here vs 50,000 in other studies).^8,11^ This more rapid development of network activity may have some benefits from a screening standpoint, as it could shorten assay times, increase throughput, and reduce costs, although ultimately, the screening needs of the user will dictate issues such as culture model and plating density. Regardless of the rate of network ontogeny, the approaches described herein can be applied to the resultant data, and in the present case, it appears that network ontogeny can be reliably predicted by using as few as three to five wells per plate by considering all of the parameters.

The use of classification techniques such as random forests and SVMs indicated that the parameters extracted from the spike trains in these experiments could be used to predict reliably the age of the culture from between the four different age categories examined. While the models performed best when all of the parameters were used to aid classification, several parameters had greater influence on the ability to predict culture age. These included the CV of within-burst ISI, CV of IBI, mean burst duration, network spike rate, and burst rate. When these approaches were used to predict between two different ages, considering all of the parameters resulted in greater accuracy regardless of age. This indicates that using multiple parameters will provide more robust discrimination of different ages (or perhaps treatments) than relying on a single or a few parameters. The greatest accuracy was achieved when predicting between larger age differences (e.g., DIV 5 vs 12) and likely reflects the relative lack of bursts, network spikes, and correlated activity in DIV 5 cultures. This is consistent with the relative lack of connectivity in DIV 5 cultures. Synaptogenesis, as reflected morphometrically by the juxtaposition of pre- and postsynaptic markers in this model, begins around DIV 6 and continues through DIV 12.^23^ Thus, the initiation of structural evidence of synaptogenesis corresponds nicely with increases in electrophysiological parameters on DIV 7, including those parameters reflecting connectivity (bursts, network spikes, and correlated activity).

The present analysis has implications for using mwMEAs for drug development or chemical developmental neurotoxicity screening. Both classification approaches used here provided higher accuracy by including more features. Traditionally, MFR has been widely utilized to describe drug- or chemical-induced alterations in network function,^2,6,11,12,18^ as it is easily extracted from the data. However, when possible, determining more features and using them collectively, rather than focusing on one or a few features, may provide greater sensitivity in detecting effects, as well as possibly facilitating drug or chemical “fingerprinting” approaches.^34^ In addition, the classification approaches used here indicate that age of a network can be reliably determined using 3–8 wells from each plate, indicating that between 6 and 16 different treatment conditions might be possible on a given 48-well mwMEA plate. We believe our study is the first to assess the important question of how to efficiently use wells on a mwMEA, suggesting that as few as three to eight wells might suffice to form a reliable pattern of activity. However, this range should be treated with caution: as can be seen from **Figure 3**, there is in increasing variance with fewer wells, and more importantly, results are likely to differ depending on the size of the effect being measured. Our findings suggest that where there are gross changes in activity patterns, fewer wells are needed. On the other hand, where changes in activity are more subtle, we would expect more replicates to be required. Our recommendation therefore is that investigators should repeat our sampling approach (**Fig. 3**) to investigate how reducing the number of wells per condition can affect the reliability of results.

One factor that influences results from MEAs is the culture-to-culture variability. Although this was not specifically addressed in the present study (typically only one plate was available from a given culture), evidence from another data set wherein three plates each were analyzed from several different cultures indicated that culture-to-culture variability is much greater than plate-to-plate variability (unpublished data). Thus, obtaining replicate values for different treatments (e.g., concentrations of a drug or chemical) across several different wells and plates from the same culture (e.g., see supplemental material in Wallace et al., 2015^35^) may be preferable to obtaining replicate values across several different cultures. It is likely that the use of a primary culture model does contribute to the culture-to-culture differences, as each culture is prepared from different animals. This may in the future be improved by the use of stem cell–derived models, which should, in theory, be more homogeneous.

In conclusion, we have described the early development of neural networks grown in 48-well mwMEA plates and found that it is qualitatively equivalent to development of network activity in single-well MEAs. Furthermore, multiparametric evaluation of the network activity parameters provides an accurate method of classifying networks by age. Together, these results indicate that neural networks cultured on mwMEAs will be a useful tool to study the ontogeny of network activity, as well as the potential for drugs, chemicals, and diseases to disrupt that activity.

## Supplementary Material

Supplementary material
