# Supplementary material for: Characterization of Early Cortical Neural Network Development in Multiwell Microelectrode Array Plates
Source: J Biomol Screen. 2016 Mar 29;21(5):510–9. doi: 10.1177/1087057116640520 (PMC4904353; doi:10.1177/1087057116640520)
Supplement: Supplementary material [file 10.1177_1087057116640520_Cotterill_Suppl.pdf]

**Supplemental Material for:**

# Characterization of Early Cortical Neural Network Development in Multiwell Microelectrode Array Plates+

Ellese Cotterill and Diana Hall, Kathleen Wallace, William R Mundy, Stephen J Eglen, Timothy J Shafer.

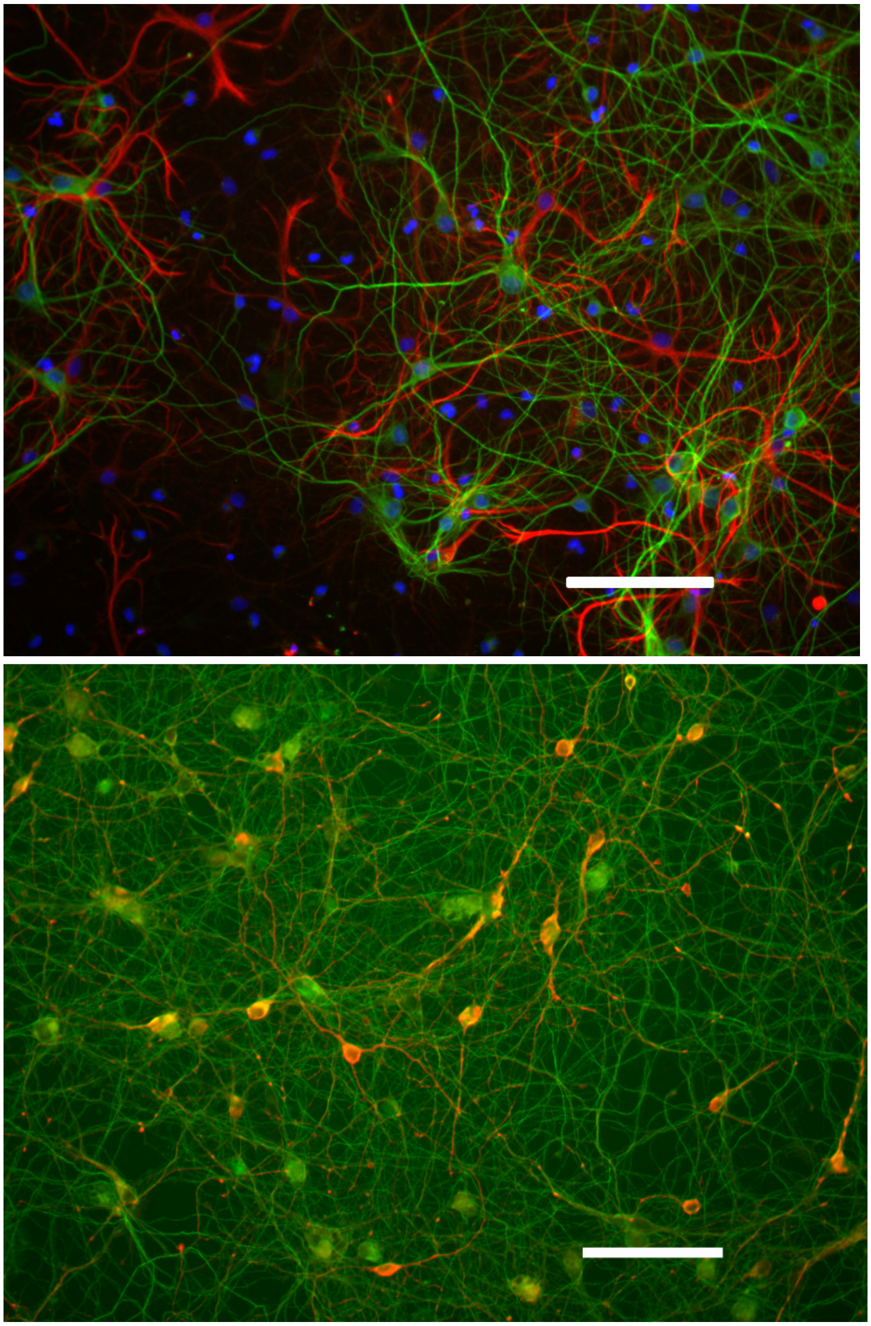

**Supplemental Figure 1. Immunocytochemical chrarcterization of cortical cultures.** Cultures were plated at a density of 20,000 cells/cm$^2$ on Corning™ Costar™ cell culture plates coated with poly-L-lysine. On DIV8 the cells were fixed in 4% paraformaldehyde and immunostained with primary antibodies to MAP2 (Millipore mab3418, 1:800), GFAP (DAKO Z0334, 1:2000), vGlut1 (Millipore mab5502, 1:400), or GABA (Sigma A2052, 1:1000) followed by Alexa Fluor secondary antibodies. **Top**) neurons (MAP2, green) and glia (GFAP, red). Nuclei are stained with DAPI. **Bottom**) glutmatergic neurons (vGlut1, green) and GABAergic neurons (GABA, red). Scale bar is 100 µm in both images. Cells were plated at a lower density than on MEAs to facilitate visualization. Oligodendrocytes are rarely observed in this culture model.

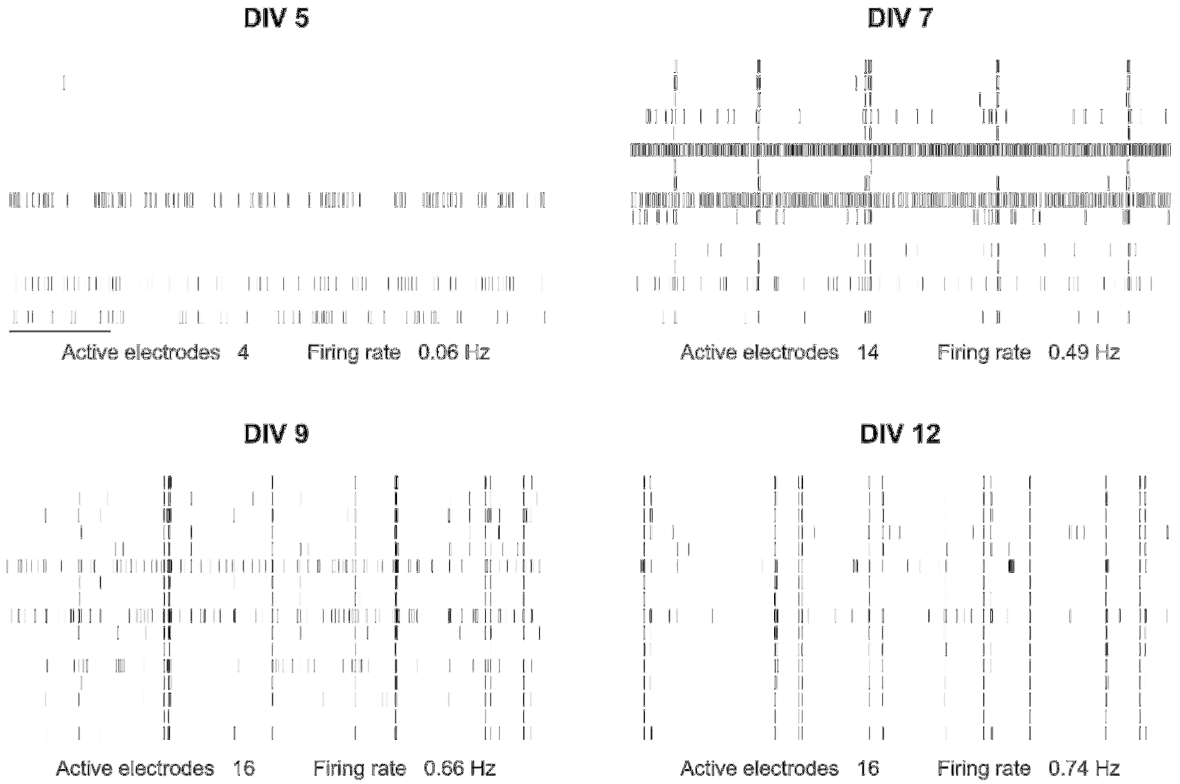


**Supplemental Figure 2: Development of network activity.** Example raster plots from the same well are shown for the four developmental ages (days in vitro, DIV) studied. Each row represents the spike train from one electrode. The scale bar for all raster plots is 60s.

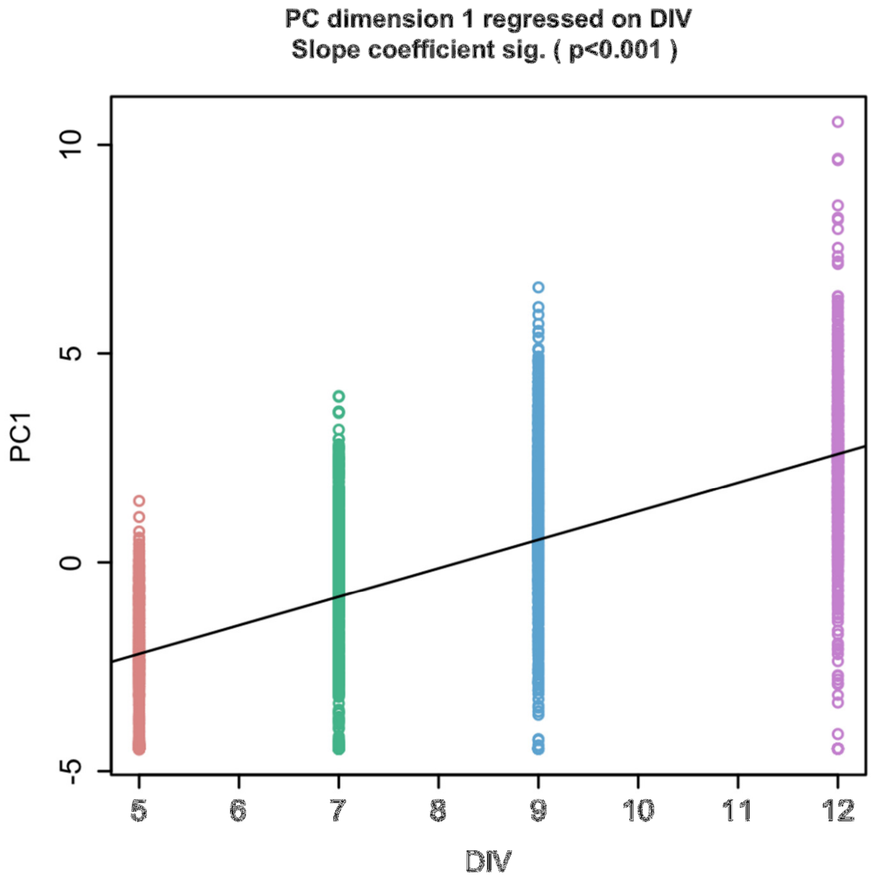


**Supplemental Figure 3: Culture age accounts for variation in PC1.** Scatter plot of projection of well level data onto first principle component (PC1), colored by DIV. Black line shows PC1 regressed on DIV, with slope coefficient p-value<0.001. PC1 increases significantly with increasing age of culture indicating that the principal mode of variation corresponds to the differences in culture age.
